# A Study of the Applicability of Existing Compact Models to the Simulation of Memristive Structures Characteristics on Low-Dimensional Materials

**DOI:** 10.3390/mi12101201

**Published:** 2021-09-30

**Authors:** Fedor Pavlovich Meshchaninov, Dmitry Alexeevich Zhevnenko, Vladislav Sergeevich Kozhevnikov, Evgeniy Sergeevich Shamin, Oleg Alexandrovich Telminov, Evgeniy Sergeevich Gornev

**Affiliations:** 1 Moscow Institute of Physics and Technology, 9 Institutskiy per., Dolgoprudny, 141701 Moscow, Russia; zhevnenko@phystech.edu (D.A.Z.); kozhevnikov.vs@phystech.edu (V.S.K.); yevgeniy.shamin@phystech.edu (E.S.S.); otelminov@niime.ru (O.A.T.); egornev@niime.ru (E.S.G.); 2 Joint-Stock Company “Molecular Electronics Research Institute” (JSC MERI), 124460 Moscow, Russia

**Keywords:** memristor, low-dimensional, compact modeling, volt–ampere characteristic, dynamic attractors, optimization

## Abstract

The use of low-dimensional materials is a promising approach to improve the key characteristics of memristors. The development process includes modeling, but the question of the most common compact model applicability to the modeling of device characteristics with the inclusion of low-dimensional materials remains open. In this paper, a comparative analysis of linear and nonlinear drift as well as threshold models was conducted. For this purpose, the assumption of the relationship between the results of the optimization of the volt–ampere characteristic loop and the descriptive ability of the model was used. A global random search algorithm was used to solve the optimization problem, and an error function with the inclusion of a regularizer was developed to estimate the loop features. Based on the characteristic features derived through meta-analysis, synthetic volt–ampere characteristic contours were built and the results of their approximation by different models were compared. For every model, the quality of the threshold voltage estimation was evaluated, the forms of the memristor potential functions and dynamic attractors associated with experimental contours on graphene oxide were calculated.

## 1. Introduction

One of the most promising solutions in the field of building neuromorphic systems is associated with a memristor [1], a nonlinear element of electric circuits, which resistance can reversibly change depending on the electrical signal entering its input. The resistive switching effect was first implemented in 2008 [2] through the atomic structure rearrangement in local regions of nanometer size in a metal–oxide–metal structure, which demonstrated the potential complementary metal–oxide–semiconductor (CMOS) compatibility, unique scalability and high switching speed of memristive devices [3]. To date, memristor research has led to the construction of complex high-performance devices capable of simulating the behavior of complex neural networks [4], building multi-level logic elements [5], and other devices for high-speed real-time operations [6].

Depending on the material of the functional layer and the design features of the memristive element, their main drawbacks vary from structure to structure, but most of them are determined by the random nature of the processes occurring inside the device [7,8,9,10,11]. One important direction in the development of memristors is the use of promising low-dimensional materials to increase the stability of device characteristics and form its new properties [12,13,14].

However, to the best of our knowledge, the current research in the field of memristive elements based on low-dimension materials does not include the study of existing memristor models’ applicability to them. In particular, the study of compact models is important because such models are used as a basis for circuit models in the development of microelectronic devices, and related to investigations of the properties of new experimental structures, for example, for investigations of dynamic attractors.

Due to the presence of a number of experimentally determined peculiarities of compact models, it is necessary to perform extraction of model parameters for each structure in order to use them. The main method used for parameter extraction is the solution of the inverse problem based on experimental measurements of the device current-voltage (I-V) curves. In general, due to the type of evolution equation, an analytical solution of the inverse problem cannot be obtained, and parameter extraction is an optimization problem.

Thus, the quality of the solution of the optimization problem in the study of memristors on low-dimensional materials determines the compact model applicability to a particular device. Therefore, introducing a function corresponding to the optimization error, it is possible to perform a comparison of the models with each other. A synthetic sample containing a set of key features of different structures, described below, was used as a simulation set to compensate for the contribution of random effects of each particular structure.

The task of evaluation of the approximation quality on a set of experimental results is important because, according to the conclusions of the “No Free Lunch” theorem [15,16], the effectiveness of models and optimization algorithms developed for them is inextricably linked to the task for which they are designed, and using them on other tasks may yield reduced effectiveness.

Examples of benchmark studies conducted to solve the optimization problem in different subject areas are presented in [17,18,19].

In this paper we performed a feature meta-analysis of memristor based on low-dimensional materials. Based on the results obtained, we conducted a study comparing linear drift models [2], nonlinear drift models [20,21,22] and threshold adaptive models [10,23,24]. In addition to the synthetic sample of the volt–ampere characteristics built on the basis of the features of the devices highlighted in various studies, the results of experiments in the study of the graphene oxide memristor characteristics, first presented in [25], are also used to investigate the descriptive power of the models. Finally, an error function is chosen and a general optimization method is proposed for the numerical experiment. Obtained results include the quality of the approximation of the volt–ampere characteristic loop, threshold voltages and the suitability of the model for the study of dynamic attractors.

The paper is organized as follows. Section 2 contains review of memristors based on low-dimensional materials and main criteria for model comparison, description of analyzed models, optimization problem statement for volt–ampere characteristic approximation, sampling of synthetic curves for model analysis, difference appearance of threshold voltages in various models, method of dynamic attractor calculation, and proposed optimization methods. Section 3 is devoted to the results of model comparison related to the approximation of synthetic and experimental volt–ampere characteristic (VAC) approximations, assessment of threshold voltages, and dynamic attractors. In Section 4 we make some concluding remarks.

At the end of the introduction, the authors would like to emphasize the novelty of the paper. To the best of our knowledge, we are the first to compare the applicability of popular compact models to the low-dimensional memristor characteristics. The article includes the requirements for a comparison based on set of test volt–ampere characteristics, proposed a general method for solving the optimization problem, and modification of the error functional to solve the optimization problem of a particular lobe of the volt–ampere characteristic. In this work we obtained the solution of the optimization problem for ten lobes of the volt–ampere characteristic of a unique shape, for the first time the study of the optimization problem for a set of close loops actually corresponding to the change of device parameters from switching to switching [11] was carried out, the threshold voltages were estimated, and for each model, for the first time for the new experimental result, an estimate of potential functions was obtained.

## 2. Materials and Methods

### 2.1. Review of Low-Dimensional Structures

The works on the study of vertical and lateral memristive structures [12,13,14,26,27,28,29] based on combinations of molybdenum disulphide (MoS_2_) with different materials demonstrate a significant variation of key characteristics. For example, the $R_{\mathrm{off}}$ to $R_{\mathrm{on}}$ ratio varies from $10^{2}$ to $10^{8}$ depending on the composition and thickness of the active layer, threshold voltages range from 0.1 V to 5 V, endurance varies from 200 cycles to more than $10^{4}$, retention up to $10^{4}\;s$ [13], and the presence of dynamic attractors [14].

Based on the results obtained from the literature analysis [12,13,14,27,28,29] and the conducted experiments, the main criteria by which it is possible to compare models are:

1. Ability to simulate a wide range of $\frac{R_{\mathrm{off}}}{R_{\mathrm{on}}}\;\sim 10^{2}-10^{8}$;
2. Ability to accurately simulate threshold voltages both for the transition from$R_{\mathrm{on}}$ to $R_{\mathrm{off}}$ and vice versa;
3. Ability to simulate a dynamic change of volt–ampere characteristic curvature near threshold voltages;
4. Ability to simulate the dynamic attractors;
5. Stability of key values of memristor simulation result on 2D structure relatively insignificant changes of the contour. In particular, maintaining a stable $\frac{R_{\mathrm{off}}}{R_{\mathrm{on}}}$
6. The result of the simulation should make sense in the original treatment of the parameters by the authors of the model, including the associated physically justified constraints.

### 2.2. Models Analysed

To simulate a memristor using compact models, it is necessary to describe the relationship between the parameters of the equations and the real parameters of the device. The focus of compact modeling is the simulation of the I-V curves of the system, the general approach involves solving the following system of equations:

(1) $$Y=F\left(x,X,Y\right)X$$

(2) $$\frac{dx}{dt}=f\left(x,X,Y\right)g\left(x,X,Y\right)$$

where X is the control signal, Y is the response, F is the function linking the input signal and the response (resistance or conductivity), g is the evolution function, f is the window function, x is the state variable of the memristor. In many cases [30] these equations are simplified to:

(3) $$Y=F\left(x,t\right)X$$

(4) $$\frac{dx}{dt}=f\left(x,X\right)g\left(x,X\right)$$

The first and most popular memristor model was proposed in [2]. The linear drift model is still relevant today and is used as the basis for other models. It is described by a system of equations:

(5) $$v=\left(R_{\mathrm{off}}\left(1-x\right)+R_{\mathrm{on}}x\right)i$$

(6) $$\frac{dx}{dt}=\frac{R_{\mathrm{on}}i\mu }{D^{2}}$$

The above equations are simple, and their influence on the appearance of the VAC can be deduced trivially from general considerations.

However, in addition to the advantages of good interpretability, computational simplicity, and stability, the model has a number of key drawbacks that make it difficult to use. The simplicity of the model is due to strong simplifying assumptions about the processes in the active layer of the memristor, which affect the accuracy of the model. Also, the model explicitly includes assumptions of switching physics that severely limit the range of approximated VACs.

The emergence of nonlinear drift models was designed to compensate for these drawbacks. An essential part of nonlinear drift models is based on [2]. Some of the first models were proposed by Joglekar and Wolf, Prodromakis, Biolek, Zha and their colleagues [31,32,33,34]. Each model was designed to correct for state evolution in the linear drift model using a window function with internal parameters. At present, there is a tendency to extend the original window functions and build generalized window functions on their basis [20,21,22], and it is the generalized nonlinear drift models that we will analyze.

Shi’s [21] nonlinear drift model is a fractional-order memristor model with the introduction of a new window function and a fractional time derivative of the state variable into the evolution equation:

(7) $${}_{0}^{C}D{}_{t}^{\alpha }x\left(t\right)=\frac{R_{\mathrm{on}}i\mu }{D^{2}}$$

(8) $$f\left(x\right)=1-{\left[a^{2}{\left(x-stp\left(-i\right)\right)}^{2}+\left(1-a^{2}\right)\right]}^{2}$$

where the parameter a lies in $\left(0,\;1\right)$, p is a positive real number, ${}_{0}^{C}{}_{t}^{\alpha }$ is the partial derivative of Caputo, defined by the formula

(9) $${}_{0}^{C}D{}_{t}^{\alpha }x\left(t\right)=\frac{1}{\Gamma \left(n-\alpha \right)}\int_{0}^{t}\frac{x\left(\tau \right)}{{\left(t-\tau \right)}^{\alpha -n+1}}d\tau .$$

Here n is an integer, n−1<α≤n, and $\Gamma \left(z\right)$ is the Euler gamma function. In the analysis of this model, α is assumed to be equal to one for simplicity.

According to the authors, models with fractional derivatives better describe physical phenomena related to nonlocality, time memory, power law and weak singularity. The proposed window function avoids problems related to stick effect, and by varying the parameter a one can achieve a change in the maximum value of the window function.

Zha’s generalized window function [20] is given by the formula:

(10) $$f\left(x\right)=1-{\left(a{\left(x-stp\left(-i\right)\right)}^{2b}+\left(1-a\right)\right)}^{p}$$

where the parameter b is a positive integer, p is a positive real number, and the parameter a belongs to the interval $\left(0,\;1\right)$. This window function solves the problem of the stick effect and scaling. The authors of the original paper propose a fine-tuning of this window function to obtain adjustable resolution in memristors using a sequence of input pulses.

A nonlinear drift model with the Li’s window function [22] was developed to overcome the stick effect, the inflexible parameter and the distorted pinched hysteresis loop problems. The window function has the following form:

(11) $$f\left(x\right)=j\left\{1-{\left[\alpha x^{3}+a^{2}{\left[x-stp\left(-i\right)\right]}^{2}+\left(1-a^{2}\right)+\beta x^{2}+\gamma x\right]}^{p}\right\}$$

(12) $$\left\{\begin{array}{c} \frac{1}{j}+{\left(1-a^{2}\right)}^{p}\geq 1,\\ {\left[\alpha +\beta +\gamma +1-a^{2}\right]}^{p}\leq 1\\ \frac{1}{j}+{\left[F\left(x_{\min}\right)\right]}^{p}\geq 1 \end{array}\right.$$

(13) $$F\left(x\right)=\alpha x^{3}+\left(\beta +a^{2}\right)x^{2}+\left(\gamma -2a^{2}\right)x+1$$

(14) $$x_{\min}=\frac{\sqrt{{\left(a^{2}+\beta \right)}^{2}+3\alpha \left(2a^{2}-\gamma \right)}-\left(a^{2}+\beta \right)}{3\alpha }$$

As the authors note, the general form of the window function allows one to adapt it to spike neural network simulations.

Another group of nonlinear drift models includes the Yang’s [35] and Simmons’ barrier models [36]. The latter model is considered to be the most accurate memristor model from Hewlett Packard (HP) Labs at the moment. Models from this group were omitted in the analysis, because in the Yang’s model the nonlinear evolution and boundary effects are introduced with window functions, and the Simmons’ barrier model is inefficient in terms of Simulation Program with Integrated Circuit Emphasis (SPICE) simulations [37].

There are also adaptive models with varying degrees of adaptivity. Such models include Yakopcic’s model [24], defined by equations:

(15) $$i=\left\{\begin{array}{cc} a_{1}x\sinh\left(bv\right), & v>0\\ a_{2}x\sinh\left(bv\right), & v<0 \end{array}\right.$$

(16) $$\frac{dx}{dt}=g\left(v\right)f\left(x,v\right)\;$$

(17) $$g\left(v\right)=\left\{\begin{array}{cc} A_{p}\left(e^{v}-e^{V_{p}}\right), & v>V\\ -A_{n}\left(e^{-v}-e^{V_{n}}\right), & v<-V_{n}\\ 0, & -V_{n}\leq v\leq V_{p} \end{array}\right.$$

(18) $$f(x,\;v>0)=\left\{\begin{array}{cc} e^{-\alpha_{p}\left(x-x_{p}\right)}w_{p}\left(x,x_{p}\right), & x\geq x_{p}\\ 1, & x<x_{p} \end{array}\right.$$

(19) $$f\left(x,\;v\leq 0\right)=\left\{\begin{array}{cc} e^{-\alpha_{n}\left(x+x_{n}-1\right)}w_{n}\left(x,x_{n}\right), & x\leq 1-x_{n}\\ 1, & x>x_{n} \end{array}\right.$$

(20) $$w_{p}\left(x,x_{p}\right)=\frac{x_{p}-x}{1-x_{p}}+1$$

(21) $$w_{n}\left(x,\;x_{n}\right)=\frac{x}{1-x_{n}}$$

Here x is the state variable, $a_{1,2}$ and b are constants, $A_{p}$ and $A_{n}$ are constants that determine the change rate of the state variable after overcoming threshold voltages, $V_{p}$ and $V_{n}$ are the absolute values of the upper and lower threshold voltages, respectively. Parameters $x_{p}$ and $x_{n}$ are restricted only to the range $\left(0,\;1\right)$.

The motivation for creating this model was the experimental confirmation of threshold voltages as well as the inability of the models [35,38,39,40] to simulate a decrease in the lobe width of the current–voltage characteristic as the average conductance per cycle increases.

The VTEAM model [23] was created by the authors as an adaptive threshold model of a voltage controlled memristor. The current–voltage relationship in this model is defined differently for each case. We use the following combination of equations for this model:

(22) $$i=\left\{\begin{array}{cc} a_{1}x\sinh\left(bv\right), & v>0\\ a_{2}x\sinh\left(bv\right), & v<0 \end{array}\right.$$

(23) $$\frac{dx}{dt}=\left\{\begin{array}{cc} k_{\mathrm{off}}{\left(\frac{v}{v_{\mathrm{off}}}-1\right)}^{\alpha_{\mathrm{off}}}f_{\mathrm{off}}\left(x\right), & 0<v_{\mathrm{off}}<v\\ 0 & v_{\mathrm{on}}<v<v_{\mathrm{off}}\\ k_{\mathrm{on}}{\left(\frac{v}{v_{\mathrm{on}}}-1\right)}^{\alpha_{\mathrm{on}}}f_{\mathrm{on}}\left(x\right), & v<v_{\mathrm{on}}<0 \end{array}\right.\;$$

where $x_{\mathrm{on}}<x<x_{\mathrm{off}}$. The VTEAM threshold model is a modification of the earlier TEAM [41] model used to simulate current-controlled memristors. The reason for creating the VTEAM model was to avoid performance and reliability problems in crossbars of threshold voltage memristors, since the voltage across all memristors is the same, and the variable resistance does not affect the procedure of switching to the high impedance state.

We previously developed a mobility modification model (MMM). This model is based on the Yakopcic’s model, which demonstrates competitive simulation accuracy and speed of the experimental VAC approximation. The model was obtained by multiplying the evolution equation by functions to account for inhomogeneities that modify the mobility of charge carriers:

(24) $$i\left(v\right)=i_{\mathrm{Yakopcic}}\left(v\right)\;\cdot \overset{n}{\underset{i=1}{\prod }}U_{i}\left(x\right)$$

(25) $$U_{i}\left(x\right)=\left\{\begin{array}{cc} \exp\left(-\frac{{\left(x-x_{i}\right)}^{2}}{2\sigma_{i}^{2}}\right), & x<x_{i}\\ 1, & x\geq x_{i} \end{array}\right.$$

Here $i_{\mathrm{Yakopcic}}\left(v\right)$ is the current–voltage relation in the Equation (15), $U_{i}\left(x\right)$ is the accounting function of the i-th inhomogeneity, is the number of inhomogeneities, is the effective position of the i-th inhomogeneity in the state space of a memristor, is the effective width of a given inhomogeneity. The motivation for creating this model was the presence of inhomogeneities in the active layer of a memristor that change the mobility of charge carriers [42].

The above equations were solved using the 4th order predictor–corrector method. The idea behind the predictor-corrector method is to use a suitable combination of an explicit and an implicit technique to obtain a method with better convergence characteristics. The explicit and implicit methods are third order Adams Methods [43], called Adams–Bashforth and Adams–Moulton methods respectively.

### 2.3. Optimization Problem

**Figure 1:**
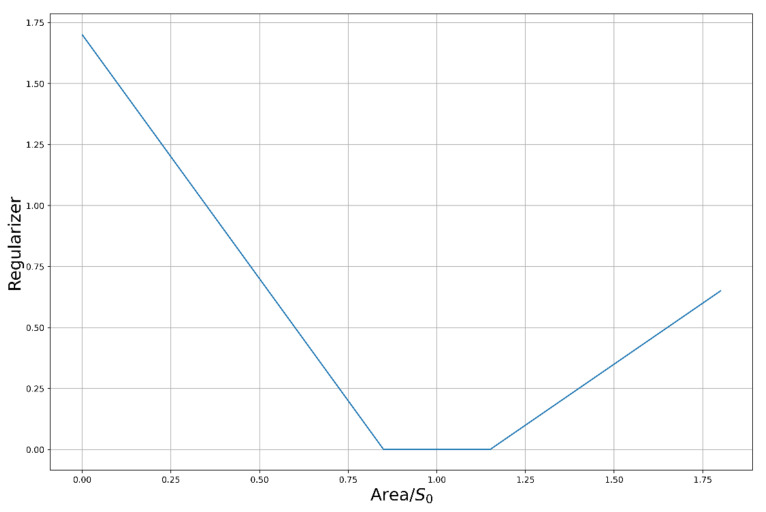
Regularizer function dependence on area of current-voltage (I-V) curve.

To compare the models with each other, it is necessary to solve the inverse problem for each model using the method of optimization of some functional. In general terms, with relation to an arbitrary criterion the functional can be written as:

(26) $$L\left(P\right)=Aim\left(P\right)+\lambda G\left(S\left(P\right),S_{0}\right)\to \underset{P}{min}$$

where P is the vector of model parameters, $Aim\left(P\right)$ is the error for the criterion, λ is regularization parameter, $S_{0}$ is area of fitted i-v curve, $S\left(P\right)$ is area of fitting i-v curve, G is the regularizer represented in Figure 1 as a function of areas.

We propose to use a regularizer related to the physical property of the curve to obtain a single solution in the parameter space of the model. This is due to the fact that the simulation result of some key features can be directly determined only by a part of the model parameters. Due to uniqueness of characteristics, regularizer based on the value of approximated contour area allows simultaneously to take into account uncharacteristic for usual contours features and to optimize values of other parameters, implementing physically adequate constraint independent from model.

### 2.4. Synthetic Sampling

Analyzing the results of both the experiment performed and those obtained in the works of other authors, we can conclude that the lowest accuracy of the obtained results refers to the region of switching between states and some of its vicinity. Moreover, the different character of switching can be observed not only between devices on different materials, but also within one series of switching. This phenomenon is connected with a set of effects: with peculiarities of experiment, with the equipment signal of registration, different nature of the functional domain effects, and so on. Thus, a significant part of the features that are possible to distinguish in the switching domain can refer not just to a structure of a certain type, but even to a specific device or even experiment. Consequently, due to the ambiguous nature of the switching region features (e.g., set voltage [26]), when comparing models, priority should be given to the pre-switching regions.

**Figure 2:**
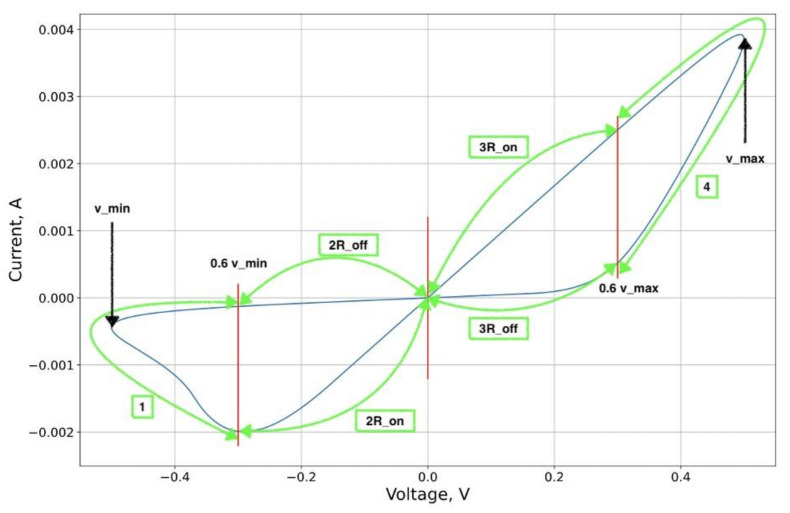
Example of volt–ampere characteristic (VAC) curve partition.

Thus, parts one and four of Figure 2 are considered indirectly, through the value of the regularizer, as a function of the area. The choice of a particular area is conditioned, among other things, by the estimation of the characteristic values of the readout voltage.

Therefore, the parts two and three of the I-V contour are used to construct the basic error function. The points over which the approximation functional was constructed were selected proportionally to the maximum voltage amplitude in the structure during the switching process.

For 100 voltage points proportional to the maximum voltage, the square of the current value difference (MSE) was calculated and averaged for the approximation result and the testing sample using the formula:

(27) $$MSE=\frac{1}{n}\overset{n}{\underset{k=1}{\sum }}{\left(I_{k}-i_{k}\right)}^{2}\;$$

where $I_{k}$ is the model current at point k, $i_{k}$ is the loop current at point k, n is the total number of points for which the error is estimated.

**Figure 3:**
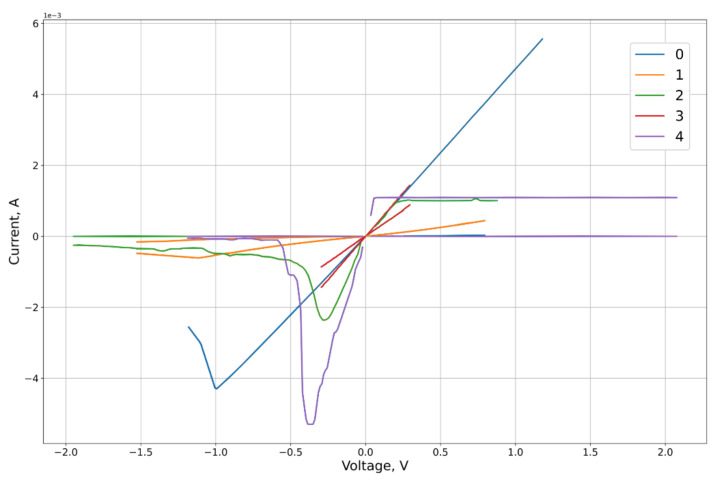
Model asymmetric elements of I-V curve.

Based on experimental contours, including [12,14,44,45,46], we designed asymmetric elements of the VAC contours (Figure 3), significantly different for right and left parts.

### 2.5. Threshold Voltages

Threshold voltages in memristor models correspond to the values of the electric field strengths, above which the memristor resistance dynamics change significantly. This effect physically corresponds to changes within the conducting regions.

Depending on the type of model, thresholds can be included explicitly or implicitly. In the VTEAM and Yakopcic’s threshold models, threshold voltages appear in the evolution equations explicitly:

(28) $$\frac{dx}{dt}=\left\{\begin{array}{cc} 0 & negative\;th.vol<v<positive\;th.voltage\\ \neq 0 & othervise \end{array}\right.$$

In the exponential drift model [47], instead of threshold voltage, a characteristic value of the electric field is accounted in the vacancy drift rate as follows:

(29) $$v\approx fae^{\left(-\frac{U_{A}}{k_{B}T}\right)}\sinh\left(\frac{qEa}{2k_{B}T}\right)=\left\{\begin{array}{cc} \mu E & E\ll E_{0}\\ \mu E_{0}e^{E/E_{0}} & E∼E_{0} \end{array}\right.$$

The threshold voltage is not specified in the linear drift model. In the nonlinear drift models based on it, the threshold voltage is not explicitly specified either. However, depending on the values of the window function parameters, one can observe a nonlinear evolution simulating the presence of threshold characteristics. In this case the evolution of the state variable occurs along the entire switching cycle.

The presence of the threshold voltages is confirmed experimentally, so it is necessary to account them in modeling. Taking into account the threshold values generate additional computational complexity, because due to the asymmetry of the I-V curve, we have to additionally determine a group of related parameters separately for the left and right lobe of the I-V curve.

### 2.6. Dynamic Attractors

Dynamic attractors are defined as dynamic equilibrium points in the state space of the memristor at fixed model parameters [48].

To find the attractors, minima of the so-called potential function of the memristor should be calculated as minima of the following function:

(30) $$U\left(x\right)=-\iint f\left(x,V\left(t\right)\right)dxdt\;$$

This formula implies that the memristor is voltage controlled and f is the right part of the evolution Equation (2).

In particular, we have previously explicitly demonstrated the possibility to obtain attractors for the Yakopcic’s and MMM models [10]. Also, the original paper by Slipko and Pershin [49] considers dynamic attractors for some nonlinear drift models with different window functions.

### 2.7. Optimization Method

As the most popular method for solving the optimization problem is a combination of multistart method for some parameters and gradient descent for the rest. Application of this approach is justified for a small number of considered switching cycles for each particular model.

The search method for an optimal solution for a set of models is determined by the complexity of each individual model, which is associated with unique parameter sets and equations complexity.

Thus, it is important to use a general global optimization algorithm. One of the well-studied global optimization methods is random search [50,51].

By random search we will imply an iterative algorithm for finding the minimum of the target function $f\left(x\right)$, $x\in X\subset {\mathbb{R}}^{n}$, with its’ step being defined as follows:

(31) $$x_{k+1}=\underset{x\in \left\{x_{k}+\zeta_{k},x_{k}\right\}}{argmin}f\left(x\right)$$

where ${\left\{\zeta_{k}\right\}}_{k=1}^{+\infty }$ is a sequence of random vectors.

A common choice of $\zeta_{k}$ is:

(32) $$\zeta_{k}=A_{k}\xi_{k}+b_{k}$$

where ${\left\{\xi_{k}\;\right\}}_{k=1}^{+\infty }$ are independent equally distributed random vectors, $A_{k}$ and $b_{k}$ are parameters (matrix and vector respectively) depending only on the results of previous iterations, e.g., in the most general case:

(33) $$A_{k}=A\left({\left\{x_{i}\right\}}_{i=1}^{k-1},{\left\{f\left(x_{i}\right)\right\}}_{i=1}^{k-1},{\left\{A_{i}\right\}}_{i=1}^{k-1},{\left\{b_{i}\right\}}_{i=1}^{k-1},{\left\{\xi_{i}\right\}}_{i=1}^{k-1}\right)$$

(34) $$b_{k}=b\left({\left\{x_{i}\right\}}_{i=1}^{k-1},{\left\{f\left(x_{i}\right)\right\}}_{i=1}^{k-1},{\left\{A_{i}\right\}}_{i=1}^{k-1},{\left\{b_{i}\right\}}_{i=1}^{k-1},{\left\{\xi_{i}\right\}}_{i=1}^{k-1}\right)$$

However, a simpler dependence is usually chosen. In the following, we will assume that:

(35) $$A_{k}=A\left(x_{k-1},f\left(x_{k-1}\right)\right)$$

(36) $$b_{k}=b\left(x_{k-1},f\left(x_{k-1}\right)\right)$$

Usually, $\xi_{k}$ is selected as normally distributed or uniformly distributed on a unit sphere.

There are theoretical results guaranteeing the convergence of this algorithm to the probability optimum under rather general assumptions, as well as estimates of the expectation of the number of steps to convergence [50,51]. For example, if we assume that functions f, A and b are continuous, and $\xi_{k}$ have a distribution with nonzero density on all space ${\mathbb{R}}^{n}$, then there is a probability convergence to a global minimum:

(37) $$f\left(x_{k}\right)\overset{\;P\;}{\to }\underset{x\in X}{min}f\left(x\right)$$

Earlier studies have been conducted [52] on estimation of expectation of number of steps τ of algorithm before achievement of accuracy >0, i.e., $f\left(x_{\tau }\right)-\underset{x\in X}{min}f\left(x\right)\leq \varepsilon$. Let $q\left(x\right)$ be the density of distribution of random variables $\xi_{k}$, $R_{\varepsilon }=\left\{x\in X\;\left|\;f\left(x_{\tau }\right)-\underset{x\in X}{min}f\left(x\right)\leq \varepsilon \right.\right\}$. Then

(38) $$\tau =O\left(\frac{\underset{x\in X}{sup}q\left(x\right)}{{\left(\underset{x,y\in X}{inf}q\left(x-y\right)\right)}^{2}}\cdot \frac{1}{\mu \left(R_{\varepsilon }\right)}\right)$$

where μ is Lebesgue measure in $R^{n}$.

We used the adaptive random search algorithm implemented in the Python programming language. The algorithm step depends on the current argument value. The step has normal distribution with zero expected value and standard deviation proportional to the absolute value of the argument.

**Figure 4:**
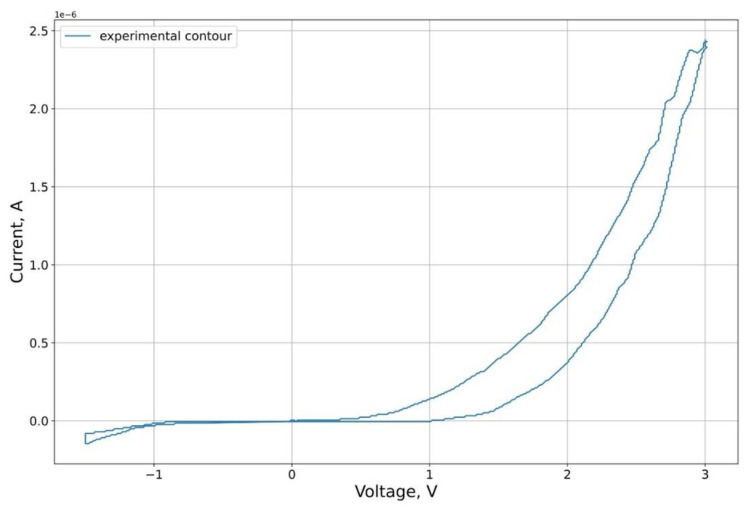
Schematic representation of the investigated structure and its volt–ampere characteristic.

As an experimental curve for the calculation, we used the results of experiments on the study of the characteristics of the graphene oxide memristor, first presented in [25]. The I-V contour of the memristor is presented in Figure 4.

## 3. Results

### 3.1. Approximation

**Figure 5:**
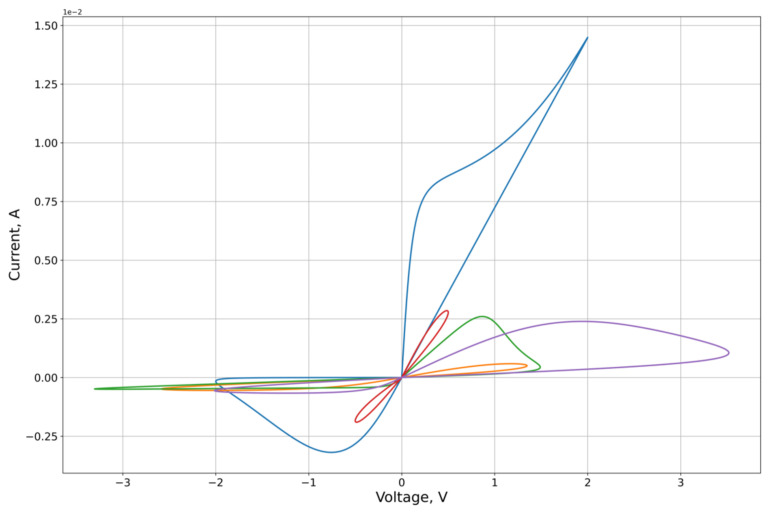
Results of the optimization of the right lobe of the synthetic sampling circuits (Figure 3) by the Shi’s model.

**Figure 6:**
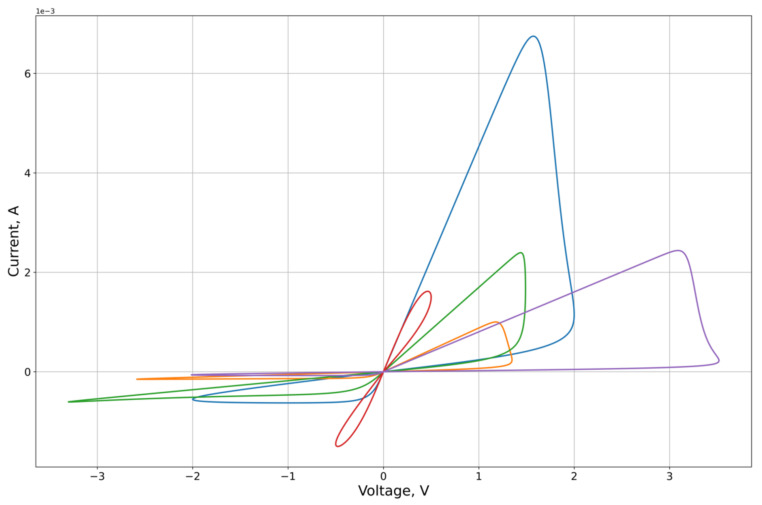
Results of the optimization of the right lobe of the synthetic sampling circuits (Figure 3) by the Zha’s model.

**Figure 7:**
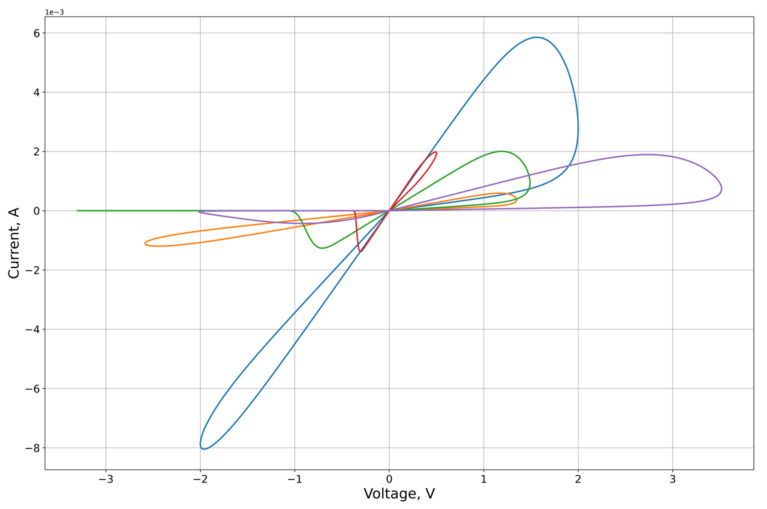
Results of the optimization of the right lobe of the synthetic sampling circuits (Figure 3) by the VTEAM.

**Figure 8:**
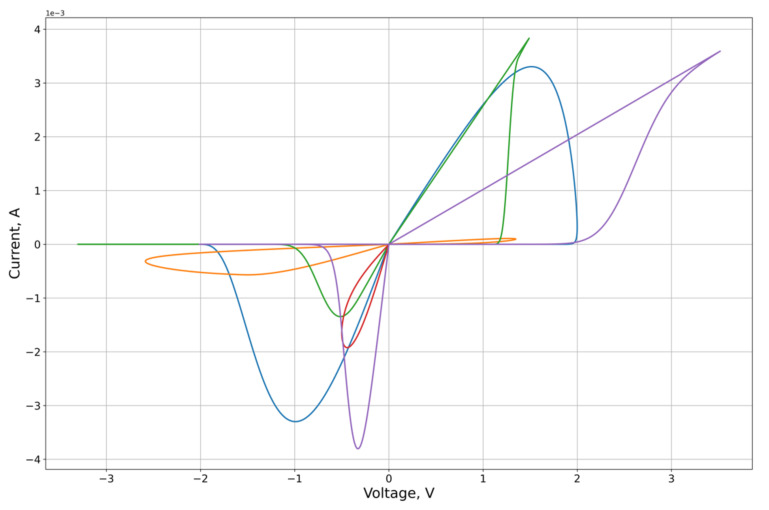
Results of the optimization of the right lobe of the synthetic sampling circuits (Figure 3) by the mobility modification model (MMM).

The feature approximation was conducted for the synthetic sample contours characteristics (Figure 3). To separate out the features of the right and left branches of the characteristics, which approximation quality we want to obtain, the calculation of the MSE on the branches $R_{\mathrm{on}}$ and $R_{\mathrm{off}}$ of the corresponding lobe was used as the error function on the criterion from Equation (26). From the results of the approximation (Figure 5, Figure 6, Figure 7 and Figure 8, Table 1) of the right and left parts of the VAC, it can be seen that the adaptive models show significantly better results to the nonlinear models. It is important to note that, despite this, for some features the nonlinear drift models show superior results, in particular Shi’s [21] model, which justifies their application to some modeling problems.

**Figure 9:**
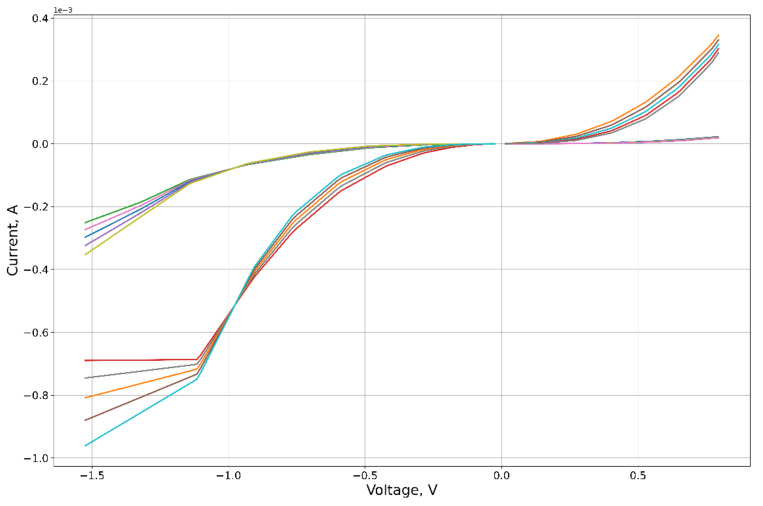
Example of curvature change of first synthetic curve.

However, in order to evaluate the optimization quality, it is necessary to make an assessment of the model stability to small changes of the test contours. To assess how sensitive the solution of the optimization problem is under such conditions, the curvature of the synthetic contours was artificially increased to 110% with a step of 2% and an insignificant change in amplitude (Figure 9).

Adaptive models remain their approximation accuracy given in the Table 1 with the increase of synthetic curves curvature. In turn, both the linear model and the nonlinear models built on its basis showed (Table 2) either no response to the change of contour curvature (which corresponds to the smooth growth of the error with curvature increase), or revealed other, significantly different from the previous state, shapes of the resulting contours (the change of metric occurred by leaps) with corresponding sharp change of model parameters.

### 3.2. Threshold Voltages

The threshold voltage was estimated within the framework of approximation by models with an explicit threshold voltage parameter. The results of the approximation are presented in the Table 3. The best result was obtained by the mobility modification model for the positive threshold voltage on the second loop: 0.87 V on the model loop and ∼1 V on the experiment. In other cases, the estimates of the threshold voltages on the loops are given, as a rule, inaccurately and differ from the experimental ones by an order of magnitude: ∼0.1 V instead of 1 V.

### 3.3. Attractors

To construct the potential function of the memristor for the experimentally obtained curve, it was approximated by the basic models. The optimal values by which the potential function is constructed are given in the corresponding tables (Table 4, Table 5, Table 6, Table 7, Table 8, Table 9 and Table 10).

**Figure 10:**
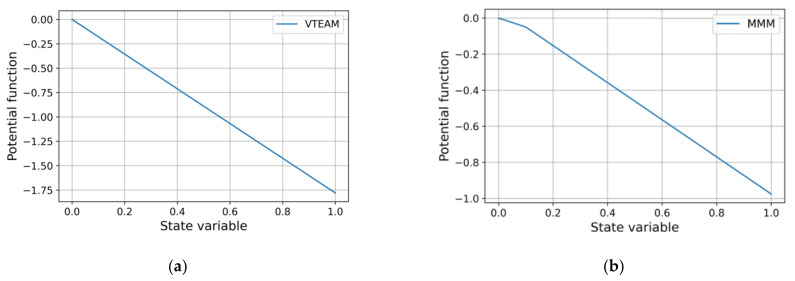
Potential functions of models with optimal parameter values: (**a**) VTEAM; (**b**) MMM.

**Figure 11:**
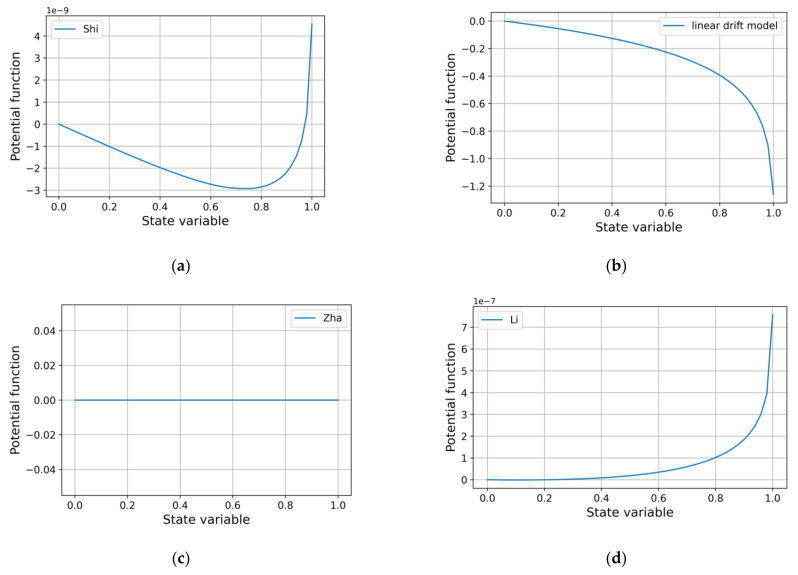
Potential memristor functions of drift models with window functions: (**a**) Shi’s; (**b**) rectangular; (**c**) Zha’s; (**d**) Li’s.

For the most accurate approximating models a similar result was obtained: the attractor is located at the right end of the region of acceptable values of the state variable (Figure 10). Less accurate models show divergent results (Figure 11): both different positions of attractors and their complete absence. The Yakopcic’s model is omitted as its potential function has the same form as that of MMM.

## 4. Discussion

In this paper we performed a feature meta-analysis of memristor based on low-dimensional materials. Based on the results obtained, we conducted a study comparing linear drift models [2], nonlinear drift models [20,21,22] and threshold adaptive models [10,23,24]. A global random search algorithm was used to solve the optimization problem, and an error function with the inclusion of a regularizer was developed to estimate the loop features. Based on the characteristic features derived through meta-analysis, synthetic volt–ampere characteristic contours were built and the results of their approximation by different models were compared. For every model, the quality of the threshold voltage estimation was evaluated, the forms of the memristor potential functions and dynamic attractors associated with experimental contours on graphene oxide were calculated.

As a result of the study, we identified several main requirements to the model. The basic ones are the possibility of simulation of a wide range of $R_{\mathrm{on}}/R_{\mathrm{off}}$, simulation of curve conductivity dynamics, stability of simulation result with respect to small perturbations of a curve. To evaluate these parameters, we performed optimization by the mean-square error of the simulation result from the approximated contour with a regularizer. To evaluate the approximation quality of different features, the optimization was performed separately for the left and right parts of the volt–ampere characteristic. The use of the regularizer was required to bound the parameters weakly related to the features of a particular area of the VAC, as well as to keep the shape of the VAC contour physically reasonable. As a result, it was demonstrated that modeling of memristor features should be based on the application of adaptive models that can, in addition to high approximation accuracy, take into account the small change in device characteristics from switching to switching.

The experimental contour of the volt–ampere characteristic was used to evaluate the accuracy of threshold voltage calculations as a result of solving the approximation problem for threshold models and to evaluate the existence of dynamic attractors as a result of the approximation by all models considered. For considered adaptive models a similar result was obtained: the attractor is located at the right end of the region of admissible values of the state variable. On the contrary, linear and nonlinear models show divergent results: both different positions of attractors and their complete absence. It is shown that for the tests given in the article the best results with respect to the chosen comparison parameters were shown by the models presented in the articles [10,23].

## Figures and Tables

**Table 1:** Square of the current value difference (MSE) values of optimization task solutions.

**Contour Number**	**Strukov Right Lobe, MSE**	**Strukov Left Lobe, MSE**	**Zha Right Lobe, MSE**	**Zha Left Lobe, MSE**	**Shi Right Lobe, MSE**	**Shi Left lobe, MSE**
1	1.05 × 10^−66^	5.33 × 10^−^^7^	2.12 × 10^−^^8^	5.03 × 10^−^^7^	1.3642 × 10^−^^9^	1.82 × 10^−^^7^
2	5.95 × 10^−8^	9.54 × 10^−^^8^	1.42 × 10^−^^8^	1.85 × 10^−^^8^	9.3538 × 10^−^^9^	1.14 × 10^−^^8^
3	8.60 × 10^−6^	5.23 × 10^−^^7^	9.37 × 10^−^^8^	1.90 × 10^−^^7^	6.3078 × 10^−^^8^	1.90 × 10^−^^7^
4	1.61 × 10^−9^	1.92 × 10^−^^9^	1.03 × 10^−^^8^	6.50 × 10^−^^9^	9.7192 × 10^−^^8^	1.36 × 10^−^^9^
5	2.82 × 10^−6^	3.04 × 10^−^^6^	1.40 × 10^−^^7^	1.70 × 10^−^^6^	1.45 × 10^−^^7^	1.79 × 10^−^^6^
**Contour Number**	**Mobility Modified Right Lobe, MSE**	**Mobility Modified Left Lobe, MSE**	**VTEAM Right Lobe, MSE**	**VTEAM Left Lobe, MSE**	**Yakopcic Right Lobe, MSE**	**Yakopcic Left Lobe, MSE**
1	8.07 × 10^−10^	1.05 × 10^−^^9^	6.53 × 10^−8^	2.64 × 10^−7^	9.37 × 10^−9^	3.29 × 10^−6^
2	1.86 × 10^−10^	1.55 × 10^−10^	1.36 × 10^−9^	1.77 × 10^−9^	1.33 × 10^−10^	1.98 × 10^−9^
3	5.85 × 10^−^8	6.11 × 10^−8^	7.17 × 10^−8^	1.83 × 10^−7^	5.97 × 10^−8^	1.51 × 10^−7^
4	3.02 × 10^−10^	2.99 × 10^−10^	1.34 × 10^−9^	8.79 × 10^−9^	3.60 × 10^−9^	3.18 × 10^−9^
5	1.39 × 10^−7^	1.85 × 10^−7^	1.40 × 10^−7^	1.22 × 10^−6^	1.39 × 10^−7^	2.12 × 10^−6^

**Table 2:** Variation of the error function from a curvature change at each point in steps of 2%.

Contour Number	Change, %	Strukov Right, MSE	Shi Right, MSE	Zha Right, MSE
1	2	1.8031 × 10^−6^	3.5255 × 10^−7^	3.1759 × 10^−7^
	4	1.9426 × 10^−6^	4.4437 × 10^−7^	3.9757 × 10^−7^
	6	2.0762 × 10^−6^	5.3765 × 10^−7^	4.9378 × 10^−7^
	8	2.2007 × 10^−6^	6.3164 × 10^−7^	5.8080 × 10^−7^
	10	2.3222 × 10^−6^	7.2599 × 10^−7^	6.6951 × 10^−7^
2	2	5.6493 × 10^−8^	2.6314 × 10^−8^	1.5371 × 10^−8^
	4	5.7224 × 10^−8^	4.7862 × 10^−8^	1.3464 × 10^−8^
	6	6.6868 × 10^−8^	6.6300 × 10^−8^	1.1857 × 10^−8^
	8	6.6492 × 10^−8^	5.1609 × 10^−8^	2.6316 × 10^−8^
	10	9.3126 × 10^−8^	1.9201 × 10^−8^	1.0540 × 10^−7^
3	2	1.5912 × 10^−7^	2.9133 × 10^−7^	1.6499 × 10^−7^
	4	1.7898 × 10^−7^	3.1641 × 10^−7^	1.4925 × 10^−7^
	6	1.9726 × 10^−7^	3.3961 × 10^−7^	1.3694 × 10^−7^
	8	2.1396 × 10^−7^	2.0479 × 10^−7^	1.6952 × 10^−7^
	10	2.2919 × 10^−7^	2.2103 × 10^−7^	1.8353 × 10^−7^
4	2	2.8724 × 10^−8^	1.1760 × 10^−7^	2.0208 × 10^−9^
	4	5.5844 × 10^−9^	8.8149 × 10^−8^	1.8848 × 10^−9^
	6	9.8905 × 10^−9^	7.1674 × 10^−9^	1.1188 × 10^−9^
	8	2.6937 × 10^−9^	1.1180 × 10^−8^	6.7628 × 10^−9^
	10	2.4394 × 10^−9^	2.9521 × 10^−8^	1.5930 × 10^−8^
5	2	2.9821 × 10^−6^	3.9269 × 10^−8^	4.4490 × 10^−8^
	4	1.2834 × 10^−6^	1.5185 × 10^−8^	9.4892 × 10^−10^
	6	2.2725 × 10^−6^	4.7225 × 10^−9^	8.9434 × 10^−8^
	8	3.6496 × 10^−6^	7.8721 × 10^−9^	1.0245 × 10^−8^
	10	4.3954 × 10^−6^	8.0405 × 10^−9^	1.8237 × 10^−7^

**Table 3:** The approximation accuracy of the threshold voltages in percent, and the values relative to which they were calculated.

Contour Number	Modified Yakopcic Left, %	Modified Yakopcic Right, %	Yakopcic Left, %	Yakopcic Right, %	VTEAM Left, %	VTEAM Right, %	Real Voltage Right, %	Real Voltage Left, V
1	99.00	85.24	90.00	100.00	99.00	92.08	1.20	−1.00
2	86.09	20.96	88.70	85.45	98.26	85.58	1.10	−1.15
3	100.00	95.56	100.00	99.61	90.63	81.79	1.40	−0.32
4	99.87	85.09	29.03	100.00	90.32	33.58	0.40	−0.31
5	84.21	65.38	99.04	88.33	78.95	90.88	3.00	−0.38

**Table 4:** Optimal values of linear drift model parameters obtained by approximation of the experimental loop.

Parameter	Value
*μ*, m^2^*/*(*V* ⋅ *s*)	1.01 × 10^−14^
*R_off_*, Ω	5.00 × 10^4^
*R_on_*, Ω	7.99 × 10^6^
*D*, m	1.00 × 10^8^
*x_start_*	0.20

**Table 5:** Optimal values of VTEAM model parameters with rectangular window function, obtained by approximating the experimental contour.

Parameter	Value	Parameter	Value
*k_on_*	−1.00	$a_{1},\;A$	2.85 × 10^−7^
*k_off_*	9.98	$a_{2},\;A$	9.2 × 10^−8^
α*_on_*	3.02	$x_{off}$	0.00
α*_off_*	0.10	$x_{on}$	0.50
*v_off_*, V	1.23	$x_{start}$	0.00
*v_on_*, V	−0.50		
*b*, V^−1^	1.21		

**Table 6:** Optimal values of the Yakopcic’s model obtained by approximation of the experimental loop.

Parameter	Value	Parameter	Value
*V_p_,* V	0.48	$\alpha_{p}$	7771
*V_n_,* V	1.43	$\alpha_{n}$	1451
*A_p_*	0.28	$a_{1},\;A$	2.2 × 10^−7^
*A_n_*	100	$a_{2},\;A$	5.84 × 10^−8^
*x_p_*	0.99	$b,\;V^{-1}$	1.44
*x_n_*	0.90	$x_{start}$	0.20

**Table 7:** Optimal values of the MMM model obtained by approximation of the experimental loop.

Parameter	Value	Parameter	Value	Parameter	Value	Parameter	Value
*V_p_,* V	0.49	$\alpha_{p}$	7771	$\sigma_{0}$	1	$x_{0}$	0.20
*V_n_,* V	1.41	$\alpha_{n}$	1451	$\sigma_{1}$	1	$x_{1}$	0.40
*A_p_*	0.28	$a_{1},\;A$	2.64 × 10^−7^	$\sigma_{2}$	1	$x_{2}$	0.60
*A_n_*	100	$a_{2},\;A$	1.28 × 10^−7^	$\sigma_{3}$	1	$x_{3}$	0.80
*x_p_*	1.00	$b,\;V^{-1}$	1.44	$x_{start}$	0.20		
*x_n_*	0.87						

**Table 8:** Optimal values of parameters of the nonlinear drift model with Zha window function, obtained by approximation of the experimental loop.

Parameter	Value	Parameter	Value
*μ, m*^2^*/*(*V* ⋅ *s*)	9.06 × 10^−^^13^	p	5.07
*R_off_* , Ω	8.45× 10^6^	a	0.25
*R_on_* , Ω	4.86× 10^4^	b	1
*D* , m	1.05 × 10^−8^		
*x_start_*	0.19		

**Table 9:** Optimal values of parameters of the nonlinear drift model with Li window function, obtained by approximation of the experimental loop.

Parameter	Value	Parameter	Value
*μ, m*^2^*/*(*V* ⋅ *s*)	3.07 × 10^−^^13^	a	0.30
*R_off_* , Ω	1.73 × 10^7^	α	1.67
*R_on_* , Ω	3.02 × 10^4^	β	0.48
*D* , m	3.34 × 10^−^^8^	γ	1.37
*x_start_*	0.07	p	3.41 × 10^−^^7^
*j*	1.46		

**Table 10:** Optimal values of parameters of the nonlinear drift model with Shi window function, obtained by approximation of the experimental loop.

Parameter	Value	Parameter	Value
*μ, m*^2^*/*(*V* ⋅ *s*)	1.39 × 10^−13^	a	0.01
*R_off_* , Ω	1.54 × 10^7^	p	3.17
*R_on_* , Ω	5.79 × 10^4^		
*D* , m	1.69 × 10^−8^		
*x_start_*	0.14		
